# Exploiting Single-Cell Tools in Gene and Cell Therapy

**DOI:** 10.3389/fimmu.2021.702636

**Published:** 2021-07-12

**Authors:** Daniel Bode, Alyssa H. Cull, Juan A. Rubio-Lara, David G. Kent

**Affiliations:** ^1^ Wellcome Medical Research Council (MRC) Cambridge Stem Cell Institute, University of Cambridge, Cambridge, United Kingdom; ^2^ Department of Haematology, University of Cambridge, Cambridge, United Kingdom; ^3^ York Biomedical Research Institute, Department of Biology, University of York, York, United Kingdom

**Keywords:** cell therapy, gene therapy, single-cell sequencing, scRNA-seq, multimodal omics, multiomics, CAR T cell therapy, disease heterogeneity

## Abstract

Single-cell molecular tools have been developed at an incredible pace over the last five years as sequencing costs continue to drop and numerous molecular assays have been coupled to sequencing readouts. This rapid period of technological development has facilitated the delineation of individual molecular characteristics including the genome, transcriptome, epigenome, and proteome of individual cells, leading to an unprecedented resolution of the molecular networks governing complex biological systems. The immense power of single-cell molecular screens has been particularly highlighted through work in systems where cellular heterogeneity is a key feature, such as stem cell biology, immunology, and tumor cell biology. Single-cell-omics technologies have already contributed to the identification of novel disease biomarkers, cellular subsets, therapeutic targets and diagnostics, many of which would have been undetectable by bulk sequencing approaches. More recently, efforts to integrate single-cell multi-omics with single cell functional output and/or physical location have been challenging but have led to substantial advances. Perhaps most excitingly, there are emerging opportunities to reach beyond the description of static cellular states with recent advances in modulation of cells through CRISPR technology, in particular with the development of base editors which greatly raises the prospect of cell and gene therapies. In this review, we provide a brief overview of emerging single-cell technologies and discuss current developments in integrating single-cell molecular screens and performing single-cell multi-omics for clinical applications. We also discuss how single-cell molecular assays can be usefully combined with functional data to unpick the mechanism of cellular decision-making. Finally, we reflect upon the introduction of spatial transcriptomics and proteomics, its complementary role with single-cell RNA sequencing (scRNA-seq) and potential application in cellular and gene therapy.

## Introduction

The crucial role that single-cell approaches play in understanding cell function has been recognised for decades. Early advances in immunology, and particularly hematopoiesis, have demonstrated the power of such approaches for ascribing functional properties to a single cell. Pioneering work by Till and McCulloch uncovered functional heterogeneity of hematopoietic stem cells (HSCs) by performing single cell-derived assays termed colony-forming unit spleen, or CFU-S, assays (1, 2). Similarly, early studies of single multipotent progenitors provided insights into the progenitor cell commitment and the development of mature immune cells, such as T and B lymphocytes (3, 4). Perhaps most transformative was the introduction of fluorescence activated cell sorting (FACS) which enabled the near-ubiquitous adaption of single-cell functional assays in immunology, hematopoiesis, and beyond (5–7).

Efforts to characterize the cellular function of single cells have fuelled an increased desire to understand detailed molecular mechanisms, but the technologies to do so in single cells have lagged substantially. The development of the polymerase chain reaction (PCR) for amplifying DNA ultimately paved the way for the first glimpse into the transcriptome of single cells (8, 9). The initial protocol for the amplification of cDNA using PCR from single macrophages was introduced by Brady et al. (10), where robust exponential amplification was achieved without disturbing the relative abundance of mRNA sequences, enabling the inspection of rare transcripts in a complex single cell-derived cDNA library. In parallel, Eberwine and colleagues developed a linear RNA amplification approach, based on the amplification of antisense RNA using a T7 RNA polymerase (11, 12). By inspecting mRNAs from single pyramidal neurons isolated from rat brains, they provided the first evidence for global molecular heterogeneity between morphologically similar cells (11).

While targeted single-cell PCR-based molecular screens revolutionized molecular biology, the low throughput and hypothesis-driven nature prevented unbiased exploratory screening. In 1991, Fodor and colleagues developed a novel photolithography-based approach for efficient synthesis of complex oligonucleotides on the microscale (13). This pioneering work would lead to the development of microarray technology where several years later, Schena et al. first applied this method for monitoring gene expression, examining the expression of 45 *Arabidopsis* genes from total mRNA (14). The following decade saw a rapid expansion of the technology, resulting in genome-wide genomic, transcriptomic and epigenetic screening using microarrays [reviewed elsewhere: (15–18)]. This ultimately enabled microarray analysis at single cell level (19), leading to insights into the molecular pathways governing cell fate (20, 21).

Microarrays, a hybridisation-based approach, assayed the known transcriptome and was therefore unsuitable for unbiased detection of novel transcripts. In 1977, Sanger and colleagues published the first genome to be sequenced (22) and soon after early generation sequencing methods began to rapidly develop (23). However, these approaches were extremely costly and time consuming (23). This opened up space for next generation sequencing (NGS) to lead to a revolution in molecular profiling, enabling low-cost, high-throughput and highly parallelised sequencing of nucleic acids. To date, a wide variety of NGS platforms have been developed [reviewed in (24, 25)] and in all cases, sheared DNA is bound to adapter sequences which are immobilised within flow cells, facilitating the synthesis of complementary DNA fragments for subsequent amplification (26). By using fluorophore-labelled nucleotides and simultaneous fluorescence readouts across the entire flow cell, the respective sequences can be determined and ultimately mapped against the reference genome (24, 27, 28). NGS for routine DNA and RNA sequencing provides multiple advantages over microarray technology, including reduced background noise, an increased dynamic range and the detection of novel transcripts (25, 29, 30).

For these reasons, NGS was rapidly adapted to a variety of model systems, including the inspection of rare cell types at single cell resolution (31–36). Tang et al. pioneered the first protocol for single-cell RNA sequencing (scRNA-seq) in single mouse blastomeres with improved performance compared to microarray-based single-cell protocols (36). Following this there has been an explosion of single-cell molecular technologies, enabling unbiased screening of the transcriptome (37, 38), genome (39, 40), DNA methylation (41), chromatin accessibility (42) and spatial resolution of gene expression (43). While these methods provide comprehensive snapshots of molecular states, their integration with cellular phenotype and function is less common and remains vital to the inspection of tissue complexity, disease progression, therapeutic intervention, and beyond. To achieve this goal, pioneering work to integrate omics protocols led to the development of several multimodal technologies. These include simultaneous screening of I) cell surface proteins and mRNA (44, 45), II) DNA methylation and mRNA (46), III) perturbations and mRNA (47), IV) DNA and mRNA (48), V) lineage tracing and mRNA, and VI) cellular function and mRNA (44, 49, 50).

Single-cell technologies have thus provided insight into a wide-range of disease mechanisms, especially in illnesses with significant heterogeneity (51), leading to a long list of potential new therapeutic options. In recent years, the fields of cellular and gene therapy have been steadily evolving for treatment of some monogenic diseases (gene therapy) and B cell leukemias (cell therapy) in particular (52, 53). However, to enable further improvements and applications to other more complex disease types such as autoimmune type 1 diabetes, key aspects such as characterizing target tissues, identifying novel targets in heterogeneous diseases and assessing efficacy of therapeutic interventions all require deeper interrogation. Recent advances in single-cell technologies are ideally positioned to address a number of these unmet needs (51).

In this review, we outline a wide range of recent technologies for screening the genome, epigenome, transcriptome and proteome of single cells and the multimodal integration of these platforms. We focus on the integration of functional cellular phenotypes with molecular profiles and emphasise the use of single-cell technologies in gene and cell therapies.

## A Golden Age for Gene Therapy - Recent Successes in Treating Monogenic Disorders

In its simplest form, gene therapy aims to cure a patient’s disease by introducing a normal or corrected copy of a gene into target cells. In 1972, Friedmann and Roblin first proposed the concept of gene therapy as a treatment for inherited genetic defects that largely affected children, many of whom experienced severe, life-threatening symptoms (54). Initially, HSC transplantation represented the primary curative option for many of these disorders, but the availability of matched sibling donors and the risk of severe graft-versus-host disease were barriers for many patients (55). To circumvent these issues, the first gene therapy clinical trials used patient-derived differentiated (T lymphocytes) or immature (hematopoietic stem and progenitor cells, HSPCs) cells that were engineered *ex vivo* to express a disease-correcting transgene (56, 57). Pioneering studies in the late 1990s and early 2000s initially reported successful treatment of adenosine deaminase-deficient severe combined immunodeficiency (ADA-SCID) and other hematological disorders (56–59); however, these successes were soon overshadowed by reports of patients who experienced significant adverse events including the development of treatment-related leukemias and severe immune reactions (60–65). Many of these unanticipated biological effects were later directly linked to the viral vectors used for transgene delivery (66, 67). Consequently, research efforts became focused on improving the safety of viral vectors (68–70) and monitoring for pre-leukemic mutations became a standard feature of treatment follow-up (71–74).

**Figure 1:**
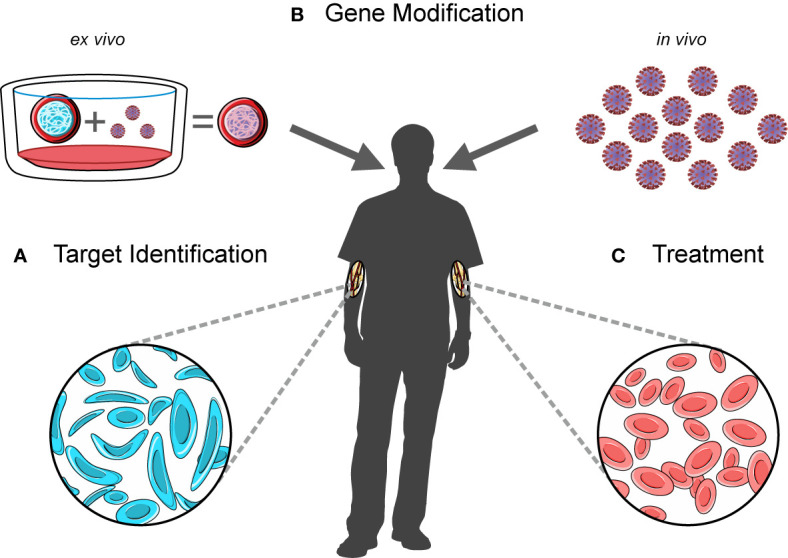
A workflow for developing and administering gene therapy. Novel gene therapy approaches involve **(A)** the identification of therapeutic targets, **(B)** an *ex vivo* gene modification step to create a transduced drug product (left) or the production of an *in vivo* product (right), and **(C)** the infusion of these products into patients following myeloablative conditioning.

Following these improvements, a number of clinical trials have demonstrated the long-term benefits achieved in individuals with various primary immunodeficiencies and monogenic blood disorders who have received gene therapy treatments (75–84). The follow-up data being reported for these patients mainly focus on disease-relevant parameters such as blood counts and overall clinical symptoms. As a result, numerous questions related to the gene therapy process still remain (**Figure 1**). For example, which HSPC populations are readily transduced during drug product creation and how does this impact outcomes? Do gene corrected terminally differentiated cells have any advantage over their non-transduced counterparts? These types of questions can best be answered using single-cell technologies. Another area of active research involves the development of *in vivo* non-viral delivery systems. These strategies include the use of nanoparticles, aptamers/oligonucleotides and extracellular vesicles to deliver transgenes or siRNAs/shRNAs (85–90). While *in vivo* treatments circumvent issues related to the isolation and manipulation of target cells, they have the potential to induce expression of transgenes or siRNAs/shRNAs in cell types that are not relevant to curing disease. High resolution single-cell transcriptomic and proteomic data will be vital in dissecting how these new treatments affect cell populations receiving the correcting vector. These types of information, especially at the level of preclinical studies, will greatly aid in the development of these technologies.

Moving beyond monogenic disorders, multi-target approaches may be useful in treating complex acquired diseases, such as cancers or autoimmune diseases like type 1 diabetes. Large-scale bulk pan-cancer genomics studies have suggested that tumors harbour an average of 4-5 driver mutations (91–94). While this represents an opportunity for the simultaneous manipulation of multiple drivers, the efficacy of this approach in individual patients depends on the specific combinations of these mutations within tumor cell subpopulations. As most genetic profiling of tumors is done using bulk sequencing, the resolution of major/minor clones and subclones becomes very difficult without the use of single-cell approaches. If individual cancers could be profiled to such high resolution, gene therapy strategies could be imagined to target genes essential to cancer cell survival (95–98) or disrupt processes such as angiogenesis that facilitate tumor growth (99–102). Combination therapies may also prove to be highly effective in some contexts (103, 104).

Type 1 diabetes is an autoimmune disease driven by loss of T cell tolerance resulting in islet autoimmunity. During disease development, insulin-producing β-cells in the pancreas are abnormally targeted by infiltrating immune cells (105). For monogenic disorders such as immune dysregulation polyendocrinopathy enteropathy X-linked syndrome where patients are at a much higher risk of developing secondary type 1 diabetes, gene therapy treatment could offer a potential cure (106). However, the genetic drivers of primary type 1 diabetes are complex and may act at the level of β-cells themselves and/or various T cell populations (105). Preclinical studies exploring the use of gene therapy to treat type 1 diabetes have clearly demonstrated the need for treatments that function on two levels - one to create or maintain functional insulin-secreting β-cells and another to protect these cells from autoimmune responses (107–110). Regardless of disease context, the overall diversity of cellular interactions driving human disease presents many challenges to the development of successful treatments. Single-cell studies can address questions pertaining to cell type interactions, disease-specific immunity, clonal dynamics of gene corrected cells and therapy-escape mechanisms, moving gene therapy forward to the next level.

## Cell Therapy as a Promising Treatment for More Complex Diseases

**Figure 2:**
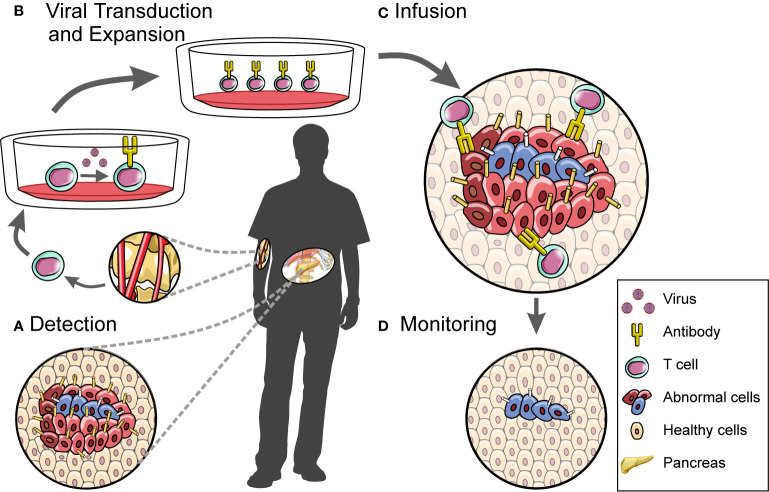
A workflow for developing and administering cell therapy. CAR T cell-based therapies involve **(A)** the discovery of disease-associated antigens which can then be used to target the cytotoxic effects of engineered CAR T cells, **(B)** the isolation and manipulation of patient-derived T cell populations, **(C)** the infusion of these cells into patients, and **(D)** downstream monitoring of disease.

While gene therapy has revolutionized the treatment of primary immunodeficiencies and monogenic disorders, other strategies may be required to treat more complex diseases. Currently, the primary standard of care for many cancers is chemotherapy, radiation therapy or, in the case of solid tumors, surgery. Immune-based treatments including cell therapy and immune checkpoint inhibitors are now being developed, already showing promise in treating refractory or relapsed patient cohorts. Cell therapy strategies involving chimeric antigen receptor (CAR) T cells have been particularly successful in the treatment of B-cell malignancies (111–113). In brief, these therapies use autologous lymphocytes with synthetically engineered antigen receptors to target tumor-specific antigens (114), thereby harnessing the immune system to trigger anti-tumor immunity (**Figure 2**). Pioneering work by several groups led to the first successful application of this technology in the treatment of B-cell malignancies (111–113), with the first therapy approved by the US-FDA in 2017 for use in B-cell acute lymphoblastic leukemia and diffuse large B-cell lymphoma (115).

Although stable remission is reportedly achieved in approximately 40-60% of patients with these B-cell malignancies (116), a number of significant barriers to increasing treatment efficacy have been identified. CAR T cell persistence and expansion has been shown to be variable between patients. Researchers have suggested that the use of less differentiated T cell subsets or T cells with an altered genetic background (for example, *TET2* disruption) during the manufacturing phase may improve outcomes (115, 117–123). However, a better understanding of the key molecular drivers of T cell expansion and persistence is required to inform future efforts to tailor the production of CAR T cells. Single-cell technologies can be used here to dissect these processes at the molecular level. In addition to increasing the overall performance of CAR T cells, another key aspect required to improve therapeutic outcomes is to control immune responses not directly mediated by CAR T cells (111–113, 124). In order to minimise these responses, a more thorough understanding of immune cell interactions must first be developed. In this context, single-cell approaches will provide the resolution required to dissect these complex systems. On a different level, selective pressures applied by anti-CD19 CAR T cells may also lead to antigen escape and lineage switching as 10-25% of patients go on to develop a CD19^-^ cancer (125). While groups reported acquired *CD19* loss-of-function mutations (126) and abnormal splicing events leading to loss of CD19 expression (127, 128), the specific origin of CD19^-^ cancer cells was not clear. A recent paper using single-cell techniques provides evidence that in at least some patients, treatment-resistant CD19^-^ cancer cells exist prior to treatment (129), underscoring the vital role of single-cell approaches pinpointing the mechanisms by which cancer cells escape treatment and informing strategies targeting refractory disease.

On the other hand, there has been relatively limited success seen in CAR T cell treatments outside of B cell malignancies, despite the development of therapeutics targeting multiple antigens simultaneously or sequentially [reviewed in (130–132)]. In solid cancers, tumor-specific antigens (TSAs) first need to be comprehensively profiled to allow for selection of appropriate candidate TSAs (133) which is especially important when dealing with heterogeneous tumors. Understanding the consequences of on-target/off-tumor effects is also essential to creating safe and effective therapies as evidenced by recent reports of adverse events experienced by patients in two separate cell therapy clinical trials (134, 135). Even once promising TSAs have been selected and tested in both animal models and early phase clinical trials, a number of other tumor-specific factors will likely interfere with the effectiveness of this treatment strategy. For example, immunosuppressive mechanisms that dampen T cell anti-tumor responses may also impact CAR T cell function. Combination therapies or further disruptions to create CAR T cells that are resistant to these immune evasion pathways may therefore become essential (136, 137). Other CAR immune cell populations such as B cells, natural killer (NK) cells and macrophages may also be useful in treating certain diseases (138–140).

In the context of diabetes, both CAR T cell and regulatory T cell (Tregs)-based treatments are currently being developed (141–146). Under normal conditions, Tregs mediate immune tolerance by expressing anti-inflammatory cytokines and dampening the inflammatory or cytotoxic responses of other types of T lymphocytes (147). While patients with type 1 diabetes have similar frequencies of Tregs compared to control individuals, it has been shown that these Tregs have reduced immunosuppressive capacity (148–150). Adoptive Treg transfers from healthy donors into patients have shown promise in preclinical models for a number of different diseases driven by immune dysregulation including type 1 diabetes (145, 151–156). However, a thorough understanding of the heterogeneous cell types that facilitate disease initiation and progression will be crucial to optimizing these treatment regimens.

## Using Single-Cell Approaches to Refine Treatment And Inform the Development of Novel Therapeutics

Although great strides have been made in gene and cell therapy, applications to a wider range of diseases requires more information. Key aspects, such as characterizing target tissues, identifying novel targets in heterogeneous diseases and assessing efficacy of therapeutic interventions require deeper interrogation and single-cell approaches are well-positioned to provide this information.

While a number of groups have begun to use single-cell approaches to dissect various aspects of CAR T cell-based therapy (129, 157, 158), the gene therapy field has not explored this to the same extent. That said, a handful of studies have used bulk sequencing approaches to examine post-transplantation clonal dynamics in a small number of patients (159–161). Biasco and colleagues used this approach to estimate transduced HSPC population size and describe the contributions of HSPC subpopulations to various stages of hematopoietic reconstitution (159, 160). Most recently, Six and colleagues addressed questions pertaining to clonal selection following gene therapy in WAS, sickle cell disease (SCD) or beta-thalassemia patients and found no indications of clonal skewing caused by insertional mutagenesis (161). While all three of these studies provide important insights into human hematopoiesis, the reliance on bulk sequencing approaches to map viral integration sites means that several key questions remain unanswerable. For example, these methods do not allow unedited cells or low abundance clones to be tracked or the effects of multiple integration sites to be assessed. Furthermore, relationships between transduced and non-transduced cells cannot be assayed. These details can only be examined using strategies that analyse single cells and their clonal progeny (162).

In contrast, studies employing single-cell technologies have already begun to deconstruct the fundamental biology behind anti-CD19 CAR T cell therapeutic outcomes. Shieh et al. used single-cell transcriptomics to identify gene signatures associated with good treatment outcomes for patients with B cell malignancies, providing insights relevant to the optimisation of CAR T cell production (157). Deng et al. used a similar approach to discover transcriptional signatures connected to both complete and poor treatment responses (158). This study also identified a novel, transcriptionally distinct cell population found specifically in the infusion products of patients who went on to develop high-grade immune effector cell-associated neurotoxicity syndrome (158). This finding demonstrates the value of single-cell approaches in generating essential information that can then be fed back into clinical practice. Another recent publication applying single-cell technologies reported that the disease-driving clone observed in one patient’s relapsed B cell acute lymphoblastic leukemia existed prior to anti-CD19 CAR T treatment (129). Taken together, these studies clearly illustrate how single cell-based datasets can provide clinically relevant insights into various aspects of the cell therapy process (**Figure 2**).

For every stage of the gene and cell therapy process, a number of important questions remain unanswered (**Table 1**). Ultimately, single-cell approaches will be instrumental both in informing our understanding of human disease and in developing the effective therapeutics required to treat them. Data generated using these methods has the potential to better inform our understanding of the numerous complex factors influencing treatment outcomes. The generation of novel targets and delivery methods for heterogeneous diseases relies on a high level of detail and the ability to map cell-cell interactions, especially for disorders with a strong immune component.

**Table 1:** Unmet needs and addressable questions in gene and cell therapy.

Prior to therapy	
What is the underlying clonal diversity for complex diseases such as cancer or diabetes? Are there tumor-specific antigens/mutations or cell susceptibilities that can be used to target various disease subclones/abnormal cell populations? Can understanding the heterogeneity of diseases refined diagnosis?	
**Isolation of cells to be edited/manipulated**	
**Gene therapy (*ex vivo* only)**	**Cell therapy**
Which HSCs are mobilized and can gene therapy outcomes be improved if this is further optimized?	Are T cells obtained from different individuals inherently different? What contributes to CAR T cell product variability?
**Manipulation of cells for therapeutic purposes**	
**Gene therapy (*ex vivo* only)**	**Cell therapy**
Are some HSPCs easier to transduce than others? Can we adjust this to improve treatment efficacy? Do HSPCs acquire mutations or epigenetic changes during *ex vivo* expansion and transduction steps?	What makes a successful T cell product? Which T cell population should be used in the production of CAR T cells? How can CAR T cells be engineered to be more specific/minimise off-target immune cell activation?
**Post-treatment follow-up**	
**Gene therapy (*ex vivo* and *in vivo*)**	**Cell therapy**
What are the clonal dynamics of edited cells over time and how does that change in relation to unedited cells? When transgenes or shRNAs/siRNAs are expressed in HSPCs, what are the molecular consequences of these changes and how do the molecular signatures of these cells compare to HSPCs from age-matched healthy controls? Can low level leukemic clones be detected prior to overt leukemias for patients? When using *in vivo* approaches, what are the consequences of gene correction or transgene expression in cells that do not usually express the gene of interest? Can *in vivo* gene therapy approaches be designed to specifically target disease-causing cells?	Which factors contribute to the toxicities associated with CAR T cells [cytokine-release syndrome (CRS), hemophagocytic lymphohistiocytosis (HLH) and/or macrophage activation syndrome (MAS)]? How can on-target, off-tumor toxicities be minimized? Which CAR T cells survive over time and are some better at targeting tumor cells than others? Are there differences between CAR T cell populations in the blood versus those present in tumor tissue? How do cancer cells (especially in solid tumors) adapt to evade targeting by CAR T cells?

## Single-Cell Multi-Omics Platforms and Their Prospect in Gene and Cell Therapy

A wide array of screening platforms have been developed to interrogate molecular states at the single cell level to give insight into tumor heterogeneity and clonal evolution of complex tissues. Here, we describe a selection of the most widely used omics tools and discuss their application in gene or cell therapy, including their potential role in addressing future clinical challenges.

### Genome

The first protocol for DNA sequencing at the single cell level, termed single nucleus sequencing (SNS), was described by Navin and colleagues (40). Comparable and reproducible detection levels of copy number variations were observed in single cell and bulk (10^6^) samples. By sequencing the genomes of 100 single monogenomic breast tumor cells and the associated liver metastatic tissue, the authors also observed substantial clonal heterogeneity (40). After FACS of single nuclei and whole genome amplification (WGA), each nucleus is sequenced in an individual flow lane. The requirement of full sequencing lanes for single nuclei limited the throughput of such experiments and consequently, several groups introduced barcoding technologies to permit multiplexing of single cells in a single sequencing lane (163–167). To address this challenge, Amini et al. developed a combinatorial barcoding approach, first using Tn5 transposome-mediated labelling followed by PCR-based indexing to yield nearly 10,000 unique barcodes (165). In turn, Vitak et al. demonstrated the efficacy of a single-cell combinatorial indexed sequencing (SCI-seq) platform by acquiring >1500 single cell genomes from a primary pancreatic ductal adenocarcinoma sample (39). To date, a multitude of single-cell sequencing platforms rely on these barcoding principles (168, 169). However, only ~32% of sequenced cells had sufficient coverage for copy-number variation (CNV) detection (39). To address this issue and avoid amplification biases of exponential WGA, Chen and colleagues developed a linear amplification protocol, significantly reducing the required resolution for CNV calling and this was further complemented by experimental and computational approaches to improve the detection of single nucleotide variants (170, 171).

Despite experimental drawbacks related to coverage, single-cell whole genome sequencing (scWGS) has enabled an unprecedented insight into clonal dynamics during tumorigenesis and normal hematopoiesis (162, 172). One notable example includes a temporal study of single human B lymphocytes that explored the evolution of mutational signatures and age-related accumulation of oncogenic mutations (173), only achievable through scWGS.

While bulk WGS studies can infer which disease-causing mutations co-occur based on average variant allele frequencies, there is the potential to group populations of cells that in reality are part of distinct clonal entities. scWGS provides a more precise overview of clonal subpopulations while also capturing information that can be used to pinpoint mutation co-occurrence and order of acquisition (174–178). This approach has been used to profile mutant clones in diseases such as childhood acute lymphoblastic leukemia, childhood T cell acute lymphoblastic leukemia and adult acute myeloid leukemia (179–181). Rare cancer cell populations missed in bulk WGS may also be detected in scWGS assays, as demonstrated by Xu and colleagues (181). Capturing this heterogeneity is essential to understanding how clones with certain mutational profiles impact disease evolution and response to treatment.

Once gene corrected cells have been infused into a patient receiving gene therapy, it is important to track the clonal evolution of these corrected cells. scWGS could be used to track these dynamics as well as answer questions surrounding whether treatment-related mutations are acquired in cells during the gene therapy process. While this method is particularly effective at identifying copy number variants and aneuploidy, technical challenges exist such as low read coverage and sequencing depth. This may significantly hamper efforts to profile single nucleotide changes in gene corrected cells. For HSPCs, bulk WGS of single cell-derived clonal cultures or colonies has bypassed these obstacles (182); however, this approach is not feasible for cell types where *ex vivo* expansion is not possible. Provided that technical challenges are overcome, scWGS represents a promising avenue to explore clonal dynamics. However, the cost for sufficient whole genome coverage in bulk and scWGS currently remains a major barrier for routine adoption.

Following cell therapy treatments, scWGS can be used to assess mutation profiles at the single cell level for highly heterogeneous tumors during the follow-up stage. This information would be particularly helpful in determining why certain patients experience disease relapse, allowing for the identification of specific clones that are either highly susceptible or resistant to CAR T cell cytotoxicity. Additionally, building a more comprehensive understanding of tumor cell clonal dynamics will be key to dissecting out subpopulations that could then be profiled with the aim of identifying new TSAs. This type of approach can be applied to any group of diseases where complex mutation profiles are expected to impact the effectiveness of treatment.

Immune receptor repertoire analysis facilitates the interrogation of clonal dynamics of the adaptive immune response and thus provides a crucial tool for immunotherapy (183). In particular, the development of VDJ-sequencing and single-cell T cell receptor (TCR) sequencing enabled robust profiling of the output of VDJ recombination, using targeted PCR and NGS (184, 185). A multitude of studies outlined the efficacy of TCR sequencing for immune cell profiling in cancer patients to help stratify patient cohorts for immunotherapy, identify the T cell repertoire in the tumour microenvironment and determine the response to PD-1 therapy (186–188). Intriguingly, computational tools have also been developed to enable retrospective VDJ profiling from global single cell sequencing data, thus negating the need for separate immune receptor profiling (157). Nevertheless, limited availability of patient tissue samples and peripheral blood can prevent identification of rare clones and sequential PCR amplification increases risk of amplification biases (189).

### Epigenome

The epigenome plays a crucial role in determining cell identity and function with chromatin organization playing a critical role in modulating gene expression and other regulatory functions (190). Chromatin accessibility is governed by the core epigenetic mechanisms of DNA methylation and post-translational modifications of histones (191). Thus, being able to screen DNA methylation, chromatin accessibility and histone modification at single cell resolution can provide crucial insight into tissue heterogeneity.

To identify open chromatin regions and characterize regulatory elements, Buenrostro and colleagues pioneered the assay for transposase-accessible chromatin using sequencing (ATAC-seq) protocol (192). In brief, this protocol leveraged the previously described hyperactive Tn5 transposase to simultaneously fragment open chromatin regions and introduce sequencing adaptors for subsequent library synthesis (164, 192, 193). While the original ATAC-seq protocol required 500-50,000 cells, the adaptation to inspect single cells soon followed. Buenrostro et al. used the Fluidigm microfluidic platform, allowing single cell capture and downstream processing of hundreds or thousands of single cells (42). Since its inception, others have developed approaches to increase the throughput of scATAC-seq to tens, or even hundreds of thousands of cells (194, 195). Illustrating its power, Sapathy et al. generated scATAC-seq profiles for over 60,000 primary human bone marrow and peripheral blood mononuclear cells (PBMC) (194). Here, the authors identified cell-type specific *cis*-elements, key transcription factor (TF) activity across a broad range of hematopoietic populations and gene activity, using aggregate accessibility of multiple *cis*-elements for a single gene. Most intriguingly, such high density of single cell clusters permits the inference of complex differentiation trajectories. Using the well-characterized development of B cells, the authors were able to reconstruct the differentiation pathway, characterize *cis*-elements of each cell type, and identify active TF programs along the entire differentiation trajectory. Unsurprisingly, scATAC-seq enabled a previously unseen insight into tumor evolution, such as the role of naïve cell types in driving tumorigenesis (194, 196, 197).

DNA methylation of cytosine residues (5mC) plays a crucial role in epigenetic regulation, including the modulation of *cis*-regulatory elements (198). In particular, DNA methylation has been implicated in gene silencing to regulate transcriptional activity during development and altering transcription factor binding (199, 200). The development of bisulphite sequencing (BS-seq) enabled unbiased, genome-wide inspection of the DNA methylome (201). To enable BS-seq at single cell resolution (scBS-seq), pioneering work by Smallwood et al. adapted the existing post-bisulfite adapter tagging protocol to derive quantitative DNA methylation signatures at up to 50% of CpG islands (202–204). Smallwood et al. and others have extensively applied scBS-seq to interrogate mouse gastrulation, human implantation, embryonic stem cells and alternative splicing at single cell resolution (203, 205–207).

The clinically-relevant utility of scATAC-seq in building a comprehensive understanding of the tumor microenvironment has been clearly shown by Sapathy et al. (194) where chromatin accessibility was mapped for more than 37,000 cells from five sets of serial basal cell carcinoma tumor biopsies. Pre- and post-PD-1 inhibitor treated samples were profiled and cell types formed clearly defined clusters, with tumor cells and non-tumor populations clustering away from one another (194). One major strength of this method is the ability to assess chromatin accessibility at specific *cis*-elements in disease-associated loci across multiple cell types. This allows for the annotation of tumor-specific, immune cell population-specific or stromal-cell specific active *cis*-elements. Aside from describing active and inactive chromosomal regions for various cell populations, scATAC-seq can also be combined with individual lentiviral integration site mapping, enabling researchers to examine where these sites fall in relation to open chromosome regions (208). This type of information can be useful in assessing whether integration of viral components in or near specific genes can be connected to robust expansion or *in vivo* persistence of CAR T cells (208). The same approaches could be used to assess how viral integration in certain chromosomal regions affects outcomes in gene therapy. These studies clearly demonstrate how this approach permits comparison of diverse cell populations that directly impact both the disease microenvironment and response to treatment.

In some diseases, therapeutic benefits may be attained through the de-repression of epigenetically silenced genes. One such example involves triggering the expression of fetal gamma-globin (HbF) to correct the pathophysiological defects associated with SCD (80, 209). One preclinical study aiming to identify a novel treatment for Fragile X syndrome used a directed DNA demethylation tool to remove methylation marks in the *FMR1* promoter region, leading to increased *FMR1* expression (210). Newly developed CRISPR/Cas9-mediated demethylation and methylation tools allow for the manipulation of the methylome (211–214). In order for these strategies to be developed into viable treatments, techniques such as scBS-seq will be required to ensure that targeting is specific and that it does not lead to outgrowth of modified cells.

Recent evidence suggests that changes in CAR T cell global methylation status may have some bearing on treatment efficacy. One study found enhanced proliferation and persistence of a dominant CAR T clone with biallelic disruption of the *TET2* gene, which encodes a demethylating enzyme (121). Another study provided evidence that decitabine treatment-mediated epigenetic reprogramming of CAR T cells led to enhanced cytotoxicity and persistence (215). scBS-seq profiling of CAR T cells in a variety of patient samples has the potential to identify novel mechanisms that play a role in determining overall treatment response.

Single-cell epigenomic screening, such as scATAC-seq and scBS-seq, can provide crucial insights into the disease microenvironment, tumor-infiltrating lymphocytes or epigenetic disruption in disease. However, the rapid technological advances in single cell epigenomics posed a new challenge – the computational analysis of large data volumes. In addition, high background noise levels, low sequencing depth and limited capture rates of single-cell epigenetic screens restricts the analytical scope of pipelines developed for bulk sequencing protocols (216). Hence, current analytical strategies leverage a pseudo-bulk approach. First, single cells are aggregated for peak calling, then individuals cells are inspected for identified pseudo-bulk peaks (217). More recently, comprehensive tools have been developed to integrate dimensionality reduction, peak calling, identification of variable peaks, motif analysis, prediction of gene association and differentiation trajectories into single pipelines (218, 219).

### Transcriptome

Single-cell RNA sequencing (scRNA-seq) is arguably the most widely applied and established single-cell molecular screening platform. Consequently, a multitude of novel scRNA-seq protocols and adaptations have been developed [extensively reviewed elsewhere: (220, 221)]. Amongst these, two major groups have emerged, primarily differing in sequence coverage to either profile full-length transcripts or sequence the 3’ or 5’ ends of captured transcripts. Picelli and colleagues pioneered Smart-seq2 for full-length transcriptomic profiling of hundreds of cells (38). Alternatively, platforms for 3’ mRNA profiling, such as Drop-seq (37) and more recently Chromium (10X Genomics) (222), utilise droplet-based microfluidic devices and unique molecular identifiers for massively high-throughput single-cell screens. This technological advance allowed profiling of tens or hundreds of thousands of cells at significantly reduced sequencing costs per cell compared to full-length profiling protocols. These high throughput techniques enable deep molecular profiling of complex tissues and are particularly beneficial for the identification of rare cell types. In contrast, full-length profiling protocols are not compatible with droplet-based approaches, thus reducing the throughput by 10- to 1000-fold at increased sequencing cost per cell (221). However, Smart-seq2 provides deeper sequencing coverage, resulting in the detection of a larger number of genes with fewer sequencing dropouts (223, 224), allowing much more robust conclusions about transcript co-expression in single cells. Increased sequencing depth also provides increased detection of low-abundance transcripts. Perhaps most useful, full-length transcript profiling also permits the detection of alternative splicing and novel transcripts (221). Taken together, both sequencing platforms provide a diverse toolbox to cover a broad range of biological questions, but it is imperative to choose the right tool for the biological question being addressed.

Multiple studies have demonstrated the utility of scRNA-seq in describing cell-cell interactions, discovering unique disease-associated cell populations, identifying minimal residual disease following treatment and even distinguishing host- versus donor-derived cells following transplantation (222, 225–228). These types of applications can easily be used to address a number of currently unanswered questions relating to all phases of the gene therapy process (**Table 1**). As a lower-cost alternative to WGS, scRNA-seq can be used to identify single nucleotide variants (SNVs) and splice variants in gene corrected cells (221, 229). Given that scRNA-seq is also particularly powerful in separating heterogeneous groups of cells (225), these datasets can be very useful in identifying genes and pathways relevant to the function of abnormal cell types that participate in the establishment of diseases such as diabetes (230, 231). In turn, this information can be employed to develop new therapeutic avenues.

Similar to its applications in gene therapy, scRNA-seq can also be used to dissect basic biological processes such as T cell development (232), aspects of which may inform the optimization of CAR T cell therapies. As discussed above, a number of studies profiling anti-CD19 CAR T cell populations before and after infusion into patients have been able to draw clinically relevant conclusions about transcriptional profiles that mark CAR T cells associated with both good and poor clinical outcomes (158, 232). scRNA-seq studies can also be used to examine interactions occurring within the tumor microenvironment between various endogenous immune cell types and CAR T cells (233).

### Proteome

The eukaryotic proteome provides the greatest molecular complexity within the genotype-phenotype paradigm. With the addition of post-translational modification, the number of functionally distinct proteins considerably exceeds the ~20,000 identified protein-coding genes (234). In addition to the complexity of the proteome, the absence of protein amplification tools has limited our ability to perform unbiased proteomic screens. Traditional hypothesis-driven approaches, such as high-resolution microscopy, flow cytometry and immunohistochemistry, have enabled protein quantification at single cell resolution (235); however, these techniques are limited by the number of screened proteins, cell throughput, and the need to know the target *a priori*. These limitations are partly addressed by mass cytometry, a high-throughput quantitative screen for up to 60 proteins using currently available protocols and a theoretical capacity of up to 120 proteins (236). The principle of mass cytometry, or cytometry by time-of-flight (CyTOF), was based on the core concept of covalent conjugation of multiple individual antibodies with unique heavy metal reporter isotopes with district ion masses (237). In brief, single cells, labelled with a complex set of reporter-conjugated antibodies, are vaporised by inductively coupled plasma to release reporter ions for analysis by time-of-flight mass spectrometry (238–240). Unique ion mass sizes permit deconvolution and ultimately the quantitative comparison of labelled proteins on individual cells.

Pioneering work by Palii and colleagues utilised CyTOF to determine the role of lineage-specific transcription factors (LS-TF) in hematopoietic lineage specification (241). By performing a temporal screen during erythropoiesis, the authors demonstrated that multipotent progenitor populations undergo gradual LS-TF changes to commit to single lineages at the single cell level. Furthermore, CyTOF has been widely applied in immune cell profiling, biomarker discovery and treatment response studies (236, 242, 243). Such findings demonstrate the power of single-cell approaches to decipher complex molecular interactions, which would otherwise be masked in bulk studies.

As previously mentioned, one of the potential risks of virus-based gene therapy is the development of an immune response targeting the delivery vehicle. A major strength of CyTOF is its ability to profile multiple cell types simultaneously, allowing researchers to create snapshots of proteins being expressed both on the cell surface and intracellularly (244, 245). With the aim of determining whether healthy donor PBMCs were reactive to viral vector components used in many gene therapy clinical trials, Kuranda et al. simultaneously profiled cytokine secretion, immune cell activation, and T cell exhaustion using CyTOF (246). Different immune cell responses were observed, some of which correlated with whether or not the donor had previously been exposed to the virus originally used to develop clinical viral vectors. These findings indicate that it may be possible to predict which patients will go on to develop vector immunogenicity (246). This type of approach can also be applied to the monitoring of immune cell interactions following CAR T cell infusion.

While CyTOF was originally developed for the screening of suspension cells, Giesen et al. pioneered imaging mass cytometry (IMC) to introduce spatially resolved mass cytometry of ~30 proteins (247). Giesen and colleagues elegantly combined traditional immunohistochemistry with laser ablation and mass cytometry, thus enabling mass cytometric screening across tissue sections with subcellular resolution. Two concurrent studies utilised IMC for screening islets and the immune cell compartment of type 1 diabetes patients at single-cell resolution (248, 249). The authors demonstrated the alterations in islet topology during disease progression and the role of T lymphocytes in β-cell destruction.

As outlined above, high-throughput single-cell phenotyping plays a crucial role in gene and cell therapy. CyTOF and other flow cytometry-based technologies, such as full spectrum flow cytometry (FSFC) and Chipcytometry, enable phenotyping of dozens of distinct cell types (250, 251). In brief, Chipcytometry utilises microfluidics to enable iterative inspection of cell surface markers, while FSFC relies on full spectral acquisition to enable parallel screening of dozens of cell surface markers (250, 251). Near limitless throughput and high capture efficiency paired with the ability to distinguish rare cell populations provides a powerful tool for immunophenotyping. Indeed, FSFC has been successfully applied to identify therapy-mediated alterations in peripheral blood mononucleocyte profiles of head and neck squamous cell carcinoma patients (252).

Despite these advances, the high cell throughput and complexity of acquired CyTOF data provides a significant computational challenge and remains a key focus area for technical development [comprehensively reviewed elsewhere: (253)]. Recent technological advances in mass spectrometry and upstream sample processing have also raised the prospect of unbiased proteomic screens. Separate work by the Slavov and Mann groups have shown a capacity to capture ~3000 and ~800 proteins per cell, respectively (254–256). At present, however, the technology is prohibitive for routine application and will require substantial development to become a powerful tool in the near future.

## Multimodal Sequencing of Complex Tissues

The development of single-cell uni-modal sequencing platforms to independently interrogate the genome, epigenome, transcriptome or proteome has raised the prospect of screening multiple components simultaneously (multimodal profiling).

**Figure 3:**
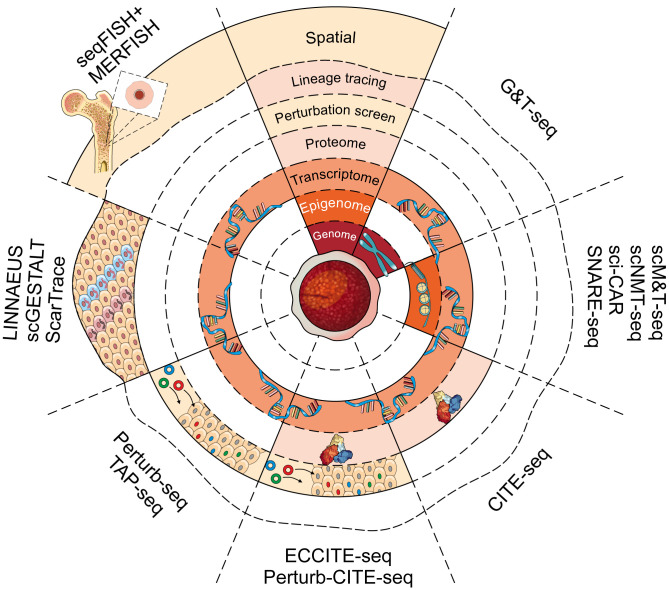
Single-cell multimodal platforms and their uses. A number of recently developed technologies can be used to assess the genomic, transcriptomic, epigenomic and proteomic landscape of a single cell. Each layer of the concentric circle represents a different molecular dimension that can be assessed using each method (from inside to outside: genome, epigenome, transcriptome, proteome, genetic perturbation, lineage tracing, spatial transcriptome). Method names are indicated along the periphery.

Numerous approaches for separating genomic DNA and mRNA from the same single cell have been proposed [various approaches extensively reviewed elsewhere: (163)]. Amongst these, the elegant G&T-seq protocol, pioneered by Macaulay et al., separates mRNA from genomic DNA by using magnetic beads and biotinylated oligo(dT) primers against poly-A tails of mRNA molecules (**Figure 3** and **Table 2**) (48). The full-length transcript profiling in G&T-seq assays provides a powerful tool for identifying alternatively spliced transcripts, fusion transcripts and expression of single nucleotide variants (SNVs) (269). The ability to associate such information with DNA copy number and structural variants at the single cell level allows unprecedented insight into the relationship of the genotype and its gene expression profiles. Nevertheless, manual separation of DNA and mRNA during the G&T-seq protocol increases sample handling, thereby limiting the throughput to hundreds of cells (269) which is further compounded by the high sequencing costs to ensure sufficient genome coverage.

**Table 2:** Multimodal single-cell tools.

Name	Modalities	Feature coverage	Throughput	Cost	References
G&T-seq	Genome + Transcriptome	Whole Genome + Whole Transcriptome	100-1000	$$$	(48)
scM&T-seq	Epigenome + Transcriptome	Whole Genome + DNA methylation	100-1000	$$$	(46)
scNMT-seq	Epigenome + Transcriptome	Whole Genome + DNA methylation + chromatin accessibility	100-1000	$$$	(257)
sci-CAR	Epigenome + Transcriptome	Chromatin accessibility + Whole transcriptome	1,000-20,000	$$	(258)
SNARE-seq	Epigenome + Transcriptome	Chromatin accessibility + Whole transcriptome	5,000-20,000	$$	(259)
CITE-seq	Transcriptome + Proteome	Whole transcriptome + 200 proteins	5,000-30,000	$$	(45)
ECCITE-seq	Transcriptome + Proteome + Perturbation	Whole transcriptome + 200 proteins + sgRNAs + VDJ recombination	5,000-30,000	$$	(260)
Perturb-CITE-seq	Transcriptome + Proteome + Perturbation	Whole transcriptome + 200 proteins + sgRNAs	5,000-30,000	$$	(261)
Perturb-seq	Transcriptome + Perturbation	Whole transcriptome + sgRNAs	5,000-100,000	$$	(47)
TAP-seq	Transcriptome + Perturbation	Hundreds of genes + Thousands of gRNAs	5,000-250,000	$	(262)
LINNAEUS	Transcriptome + Lineage Tracing	Whole transcriptome + Lineage	1,000-10,000	$$	(263)
scGESTALT	Transcriptome + Lineage Tracing	Whole transcriptome + Lineage	1,000-10,000	$$	(264)
scarTrace	Transcriptome + Lineage Tracing	Whole transcriptome + Lineage	1,000-10,000	$$	(265)
seqFISH+	Transcriptome + Spatial	Up to 10,000 genes + Subcellular location	Thousands (limited by field of view and imaging time)	$$$	(266)
MERFISH	Transcriptome + Spatial	Up to 10,000 genes + Subcellular location	Thousands (limited by field of view and imaging time)	$$$	(267, 268)

Whole genome sequencing (WGS) approaches provide a crucial tool for characterizing genomic abnormalities in primary tumors (270). Zhu et al. recently applied G&T-seq to a subset of lymphovascular invasive cells, isolated from a breast cancer patient (271), describing the relationship between RNA and CNV clones and outlining multiple functionally distinct clones and their role in metastatic dynamics. This illustrates the power of G&T-seq to uniquely integrate genomic abnormalities with transcriptional consequences, potentially of substantial utility in deciphering tumor heterogeneity and intra-tumoral clonal dynamics post CAR T therapy.

Existing epigenetic single-cell assays have also been adapted to enable multimodal approaches (**Figure 3** and **Table 2**). For example, Angermueller et al. adapted the existing principles of G&T-seq by introducing a bisulfite treatment step which allowed DNA methylation profiles and gene expression to be obtained from the same cell (scM&T-seq) (46). A more recent adaptation to the scM&T-seq protocol introduced chromatin accessibility as the third dimension for simultaneous single-cell nucleosome, methylation and transcription sequencing (scNMT-seq) (257). Here, a methyltransferase is used to label accessible DNA prior to scBS-seq. Such labelling permits downstream computational deconvolution of DNA methylation and chromatin accessibility profiles (272). To date, scM&T-seq and scNMT-seq have provided intriguing insight into stem cell biology and mouse gastrulation. For instance, pioneering work by Argelaguet and colleagues described the role of epigenetic priming at lineage-specific enhancers during lineage commitment (205). A second pioneering study revealed that changes in DNA methylation drive increasing transcriptional heterogeneity during stem cell ageing (273). These studies demonstrate the impact of a multi-modal scNMT-seq for characterising the role of the epigenome in complex tissues and biological processes, including the underlying cellular heterogeneity.

Taking into account the role of DNA methylation in driving autoimmune defects, age-related diseases and tumorigenesis (274, 275), scNMT-seq can provide a powerful and versatile tool for uncovering novel therapeutic avenues. These principles can also be applied for assessing the extent to which normal tissue function can be restored following corrective gene therapies. Similarly, multimodal epigenetic and gene expression profiling can provide a valuable tool for characterizing the tumor microenvironment and its interaction with CAR T cells to increase therapeutic efficacy. However, the relatively low-throughput of scNMT-seq can limit the coverage of large, complex tissues.

To determine the impact of *cis*- and *trans*-regulatory elements on gene expression profiles, collecting chromatin accessibility and gene expression profiles from the same cell are of paramount importance. Cao et al. pioneered sci-CAR to simultaneously perform nuclear scRNA-seq and scATAC-seq (258) by adapting previously established principles of single-cell combinatorial indexing to barcode mRNA and open chromatin regions from single nuclei extracts. Shortly thereafter, Chen and colleagues developed SNARE-seq for performing simultaneous gene expression and chromatin accessibility profiling (259). In contrast to sci-CAR, SNARE-seq utilized the high-throughput Drop-seq platform to incorporate single nuclei and adapter-coated beads. Upon nuclei lysis within each droplet, released nuclear RNA and chromatin fragments bind to the uniquely barcoded beads allowing connectivity of ATAC-seq and RNA-seq profiles of individual cells. Furthermore, SNARE-seq enabled significantly improved capture of chromatin fragments and improved the transcript sequencing depth (259). That said, the potential of SNARE-seq is partially restricted by the complexity of downstream data analysis and this prompted the development of integrated analysis pipelines, such as Signac (218) and the Chromium Single-Cell Multiome ATAC + Gene Expression platform. The simplification of the sample preparation process and analysis pipelines will be required to facilitate the wider adoption of multi-modal epigenetic and gene expression screening.

A vast array of computational tools has been developed for the analysis of unimodal single cell data. For instance, advances in dimensionality reduction, clustering and algorithms for identifying marker genes, constructing lineage trajectories and batch correction contributed greatly to current widespread access to scRNA-seq analysis tools (163). The assembly and curation of key tools into unified analysis pipelines, such as Seurat, SCRAN or SCANPY, has enabled bench-trained scientists to independently analyse scRNA-seq data (276–279). Datasets from multimodal analysis with distinct cellular dimensions inherently do not share common features (280), making data integration across distinct modalities from the same cell a profound and novel computational challenge. To integrate multiple modalities collected from the same cells into a single reference describing cell identities, Hao et al. developed a Weighted Nearest Neighbour (WNN) framework (281). In brief, WNN utilises nearest neighbour analysis and computes modality weights to derive a single landscape, reflecting the similarities of all modalities. The increased adoption of single-cell multimodal screens provides another computational challenge - the integration of multimodal data across distinct experiments, platforms and batches. While multiple strategies to integrate and batch-correct unimodal scRNA-seq datasets have been proposed (278, 282), their applicability to multimodal datasets is limited. To overcome this limitation, Stuart and colleagues adapted canonical correlation analysis and L2 normalisation to derive anchors for data integration (283). To enable integration in a variety of experimental settings, several anchoring methods have been proposed [reviewed in (284)]. Nevertheless, the rapidly expanding landscape of novel multimodal screening technologies continues to require bespoke analytical approaches and recent developments in multimodal data analysis are expansively reviewed elsewhere (163, 280).

Overall, technological advances have resulted in an unprecedented proliferation of novel single-cell molecular assays. Intriguingly, the capability of incorporating such approaches to acquire multiple elements from single cells has allowed the interrogation of the direct relationship of multiple molecular dimensions. Such extensive single-cell profiling is particularly beneficial for application in future cell therapies where the interrogation of tumor infiltrating lymphocytes and tumor microenvironments will provide a crucial component for target discovery and monitoring of therapeutic efficacy. Due to the heterogeneous nature and shifting clonal dynamics of malignant tissues, single-cell approaches are of paramount importance for the development of effective cell therapies.

## Multimodal Single-Cell Approaches Integrating Functional and Molecular Data

Simultaneously acquiring functional and molecular readouts from the same cells have historically represented an experimental challenge, as omics profiling tools typically result in destruction of the target cell. This is particularly challenging when the functional state of a cell is determined by a retrospective assay, thereby making its prospective isolation and molecular characterization impossible. Hence, most technical developments that combine functional and molecular multimodal approaches have focused on capturing cellular function prior to a destructive single-cell assay.

### Transcriptome and the Cell Surface Proteome

One of the first applications of multimodal omics technologies arose from the desire to connect cell surface phenotypes with gene expression profiles. Several well-characterized biological systems, particularly immune cell subtypes and hematopoiesis, have benefited from in-depth characterization of cell surface markers for a variety of functionally distinct cellular populations (285). As a result, quantitative phenotypic information of selected cell surface markers can permit inference of cellular function. Fluorescence-activated cell sorting (FACS) in combination with index sorting allows simultaneous recording of cell surface protein levels prior to deposition in lysis buffer for downstream destructive molecular assay, such as the Smart-seq2 protocol for gene expression profiling (38). The application of such approaches has allowed the linkage of stem cell function with global molecular profile for the first time and provided numerous insights into our understanding of transcriptional heterogeneity throughout hematopoiesis (44, 285–287).

Strategies involving index sorting and downstream scRNA-seq are particularly powerful when combined with functional outcome analyses. Wilson et al. and others have shown how these methods can be applied to understanding the heterogeneity inherent to many normal tissues and identifying features that differentiate normal and disease-causing cell types (44, 287–292). These methods would be particularly useful in linking T cell function to distinct gene expression profiles, allowing for the identification of subpopulations of cells that are associated with specific clinical outcomes.

Nevertheless, isolation strategies of functional cell types frequently do not achieve homogeneity and contaminating cells cannot be fully excluded from destructive molecular assays. This is in contrast to selective single-cell functional assays that can distinguish truly functional cells from contaminants, meaning that cellular heterogeneity is often the first to be identified (i.e., they drop out of the assay and do not generate a confusing data point) (293). Furthermore, cell isolation by FACS requires prior knowledge of distinct cell types, thereby precluding the discovery of novel cell types. In addition, index-sorting FACS-based approaches are not compatible with droplet-based high-throughput sequencing platforms. To overcome these limitations, Stoeckius et al. pioneered CITE-seq (cellular indexing of transcriptomes and epitopes by sequencing, **Figure 3** and **Table 2**) (45). Here, antibodies against cell surface proteins of interest are labelled using unique oligonucleotide barcodes. Antibody-labelled cells are subjected to the Drop-seq protocol, encapsulating single cells in droplets containing beads to introduce unique cellular barcodes to mRNA and the antibody-derived tags (ADTs). Subsequently, ADT counts are used to quantify antibody-bound cell surface proteins and provide a link to the corresponding single-cell gene expression profiles. Consistent surface proteome quantification and resolution were achieved compared to traditional flow cytometry approaches, while providing a theoretically unlimited scope for antibody multiplexing (45).

The application of CITE-seq in tumor microenvironment biology has been noted previously (294, 295). Praktiknjo et al. screened healthy and tumor-bearing mouse salivary glands, including the immune compartment of the tissue (295). By performing CITE-seq, the authors were able to construct a comprehensive gene expression atlas and simultaneously recorded a comprehensive set of 63 immune-specific cell surface proteins. Most notably, they derived a comprehensive cell atlas of the tumor microenvironment, using gene expression profiles and quantification of cell surface proteins, underscoring the utility of CITE-seq in the discovery of novel tumor-specific cell surface antigens for cell therapy. By linking surface protein quantification with gene expression profiling at single cell resolution, CITE-seq can identify novel antigens associated with specific clones within heterogeneous cancer tissues, ultimately raising the prospect of a broader spectrum of effective cell therapies. The efficacy of multimodal single-cell screens, such as CITE-seq has been particularly evident throughout the scientific response to the COVID-19 outbreak. Combined efforts to screen >780,000 single PBMCs from COVID-19 patients and healthy donors using CITE-seq revealed the immune response to COVID-19 infections and its role in disease pathology (296). Such studies provide a prominent example how single-cell multiomics can provide rapid insight into previously unknown diseases and help inform the development of effective therapeutics.

### Perturbation Screens

Large-scale perturbation screens have previously provided unprecedented insights into gene functions and their role in complex biological mechanisms (297). The advent of CRISPR/Cas9 has revolutionized our ability to conduct high-throughput perturbation screening and multiple groups have now developed multimodal single-cell perturbation screens, combining CRISPR technology with scRNA-seq (47, 298–301). In Perturb-seq (**Figure 3** and **Table 2**), a pool of barcoded single-guide RNAs (sgRNAs) is constructed against a set of 24 transcription factors and transduced cells are subjected to high-throughput droplet-based sequencing, whereby unique cell barcodes are also introduced. The dual barcoding approach allows connection of single-cell gene expression profiles with a respective perturbation. Such single-cell CRISPR screens and their ability to interrogate transcriptional consequences of perturbations provided a novel method to assess the functional effectors of complex biological mechanism and tissues (301, 302). Of note, Jin et al. demonstrated the application of Perturb-seq in an *in vivo* setting (303). To interrogate the underlying molecular mechanisms driving autism, the authors introduced a guide RNA pool against risk genes to the forebrain of a developing embryo *in utero*. The progeny of perturbed cells was then collected at P7 for downstream scRNA-seq analysis, providing key insights into the molecular mechanisms of neocortical cell types.

Perturb-seq can be very useful in trying to understand larger pathways that integrate multiple signals. For example, Adamson et al. used Perturb-seq to understand how activation of the unfolded protein response (UPR) differed between individual cells (301). This type of data has the potential to disentangle larger signaling networks, all of which is important for understanding complex processes such as immune responses.

Despite the demonstrated efficacy, application of Perturb-seq is limited by the sequencing depth of high-throughput approaches. Acquired data is subject to significant background noise and low-abundant transcripts are frequently missed (47, 298). Furthermore, the multiplicity problem of combining multiplexed perturbations with single-cell gene expression profiles poses a computational challenge. Schraivogel proposed an intriguing adaptation, termed targeted Perturb-seq (TAP-seq) (262). By performing targeted amplification of a selected set of genes prior to sequencing, the cost and analytical complexity could be significantly reduced. This approach provides a powerful tool for screening cellular pathways with defined genetic biomarkers. In the context of cell therapy, TAP-seq could thus provide a cost-effective tool for identifying underlying molecular mechanisms of immune cell evasion of CAR T therapy.

There have been a wide variety of additional approaches to integrate single-cell perturbation screens with the surface proteome of the same cell. Most notably, Mimitou et al. proposed ECCITE-seq (260) and Frangieh et al. described Perturb-CITE-seq (261). In brief, Mimitou et al., adapted the existing CITE-seq protocol by introducing addition oligonucleotides against unique sgRNA identifiers to cellular barcoding beads. Thus, sgRNA, transcripts, antibody-oligonucleotides and up to 2 other parameters can be recorded for individual cells (260). More recently, Frangieh et al. proposed Perturb-CITE-seq to provide a scalable solution for Perturb-seq with simultaneous screening of cell surface proteins (261). Here, the authors demonstrated the benefits of Perturb-CITE-seq by identifying molecular pathways driving immune evasion of a melanoma cell line against primary tumor infiltrating lymphocytes (261). Overall, the ability to connect gene expression profiles and the cell surface proteome from single cells under perturbation provides a comprehensive characterisation of complex molecular systems. As demonstrated by Frangieh et al., such technologies can help identify and characterize immune evasion drivers and ultimately reveal novel targets that might lead to enhanced therapeutic potency of immunotherapies.

### Clonal Tracking and Lineage Tracing

Recent work by Lee-Six et al. outlined the application of whole genome sequencing (WGS) approaches to establish the clonal dynamics of human HSPCs (182). The authors isolated single HSPCs from a healthy donor and were able to retrospectively reconstruct the phylogenetic tree of single cell-derived colonies, based on a broad set of shared or unique acquired somatic mutations. By simultaneously screening mature cells isolated from peripheral blood samples of the same individual, Lee-Six et al. were able to infer the progeny and extended relatedness of stem cell clones. Using this approach in a 59 year old human, the authors could map all the way back to the most recent common ancestor for blood and buccal epithelium, observed an early expansion of the stem cell compartment and confirmed hematopoietic activity of a large number of diverse HSC clones estimated to be between 50,000 and 200,000 actively contributing HSCs (162, 182).

This technique could be powerfully applied to gain insight into the clonal dynamics of HSCs used in gene therapy. Careful patient monitoring must be undertaken to ensure therapeutic efficacy and restoration of normal tissue function. As multipotent cells provide the most common target for gene therapies, gene corrections can significantly impact the clonal dynamics of the target tissue. Intriguingly, previous efforts to track therapeutic efficacy of corrective therapies large depended on monitoring progeny cells, their homeostatic function and particularly the proportion of target cells expressing the desired gene edit (159, 304). However, such approaches do not provide sufficient resolution to fully characterize clonal dynamics of corrected cell types and their impact on homeostatic tissue function. WGS of single cell-derived colonies allows to monitor naturally occurring somatic mutations in multipotent cells and their progeny to establish their relationship and infer clonal dynamics of single cells (162). When applied to a pool of edited cell and mature cell progeny post-gene therapy, such approaches can provide a direct insight into therapeutic efficacy and long-term tissue health.

In contrast, upfront labelling of target cells followed by temporal tracking of their progeny can reveal patterns of clonal evolution. Here, the advent of routine and cost-effective sequencing also revolutionised lineage tracing, providing a compelling alternative to traditional imaging-based approaches. In the context of diabetes, lineage tracing has been used to track the various cell types which originate from pancreatic progenitor cell populations (305–307) and identify cell types that are able to transdifferentiate into insulin-secreting cells (110, 308, 309). High-throughput screening at single cell resolution and integration into multimodal approaches greatly expand the scope of lineage tracing (310). While fluorescent tags limit the capacity of parallel barcoding, DNA sequence complexity provides a scalable barcoding approach. In principle, unique DNA barcodes are first introduced into a large population of target cells. Subsequently, amplification of the unique set of DNA barcodes in cell progeny can be used to compute lineage phylogenies (311, 312). A prominent barcoding approach relies on CRISPR/Cas9-mediated dynamic lineage tracing. Here, CRISPR/Cas9-mediated double-stranded breaks are introduced at defined genomic loci (313). The resulting insertions and deletions (indels) create unique cellular barcodes, which evolve over time. By sequencing such regions, the mutational patterns can be used to establish phylogeny and clonal evolution. Multiple groups have independently pioneered such CRISPR/Cas9-based lineage tracing approaches, which predominantly differentiate in the number of loci used to store lineage barcodes (263, 265, 314–318). Of note, using genome editing of synthetic target arrays for lineage tracing (GESTALT), McKenna et al. were able to trace and reconstruct early developmental pathways in a whole organism.

Dynamic lineage tracing protocols outlined above have been integrated in multimodal screens to link cellular progeny to their respective gene expression profiles, including single-cell GESTALT (scGESTALT), linear tracing by nuclease-activated editing of ubiquitous sequences (LINNAEUS) and ScarTrace (**Figure 3** and **Table 2**) (263–265). Raj et al. integrated the underlying principles of GESTALT with scRNA-seq to simultaneously acquire lineage information and gene expression profiles of the same cell (264). Instead of targeted sequencing of genomic DNA, scGESTALT relies on sequencing of expressed transgenes, which encode the unique cellular barcode. The use of droplet-based high-throughput gene expression thus provides cell type information, otherwise lost in previous lineage tracing protocols. Intriguingly, the LINNAEUS and ScarTrace protocols introduce barcodes in fluorescent transgenes to allow monitoring of successful integration of cellular barcodes. Thus, providing a crucial quality control mechanism prior to performing computational- and capital-intense sequencing (263, 265).

While prospective lineage tracing is not possible in humans, the use of these techniques in preclinical studies has the potential to unlock cellular relationships that are relevant to understanding cell origins in normal and diseased tissues. Furthermore, lineage tracing may also be used to link immature immune cell types to their immunologically active terminally differentiated counterparts. This could feed into refinements of CAR T cell production protocols for example, allowing for the selection of specific populations with maximal effector function (117).

Nevertheless, these multimodal lineage tracing technologies are currently in their infancy and a variety of experimental and computational limitations require attention. Shallow sequencing depth of high-throughput approaches can prevent barcode detection and CRISPR/Cas9-induced cell toxicity has recently been described, thus potentially disrupt the effective construction of phylogeny or distort separation of cell types (310, 319, 320). Furthermore, Spanjaard et al. noted the probability of double scarring, whereby a subset of non-homologous end joining-mediated errors have a higher probability of occurring (263). Thus, if not excluded, high-frequency scars can result in false inference of lineage relationship. To address the issue of barcode duplications and noise, Zafar et al. recently proposed a novel analytical pipeline for improved lineage tree reconstruction and integration of separate single-cell lineage tracing experiments (49). While these advances are promising, further computational innovation will be of paramount importance for the adoption of single-cell lineage tracing in gene and cell therapy developments.

## Introducing Spatial Resolution in Gene and Cell Therapy

Single-cell sequencing technologies and their multimodal integration continue to push the boundaries of understanding the mechanisms governing complex tissue organization. However, such single-cell screening protocols are largely based on removing the cells and destroying them, typically discarding any spatial information of the underlying tissue from which they were extracted. The crucial role of cellular location and spatial gene expression throughout early embryogenesis has been widely recognized (321). Similarly, cellular location in heterogeneous tumors and the surrounding tumor microenvironment are vital to cell function (322). Therefore, resolving spatial dimensions and linking these with gene expression profiles to infer gene function and cell identity can help us understand disease pathology and complex tissue function. Here, we discuss selected technological developments in spatial transcriptomics and their prospect in the development of novel cell and gene therapies [spatial omics protocols are comprehensively described elsewhere: (321, 323)].

The development of fluorescence *in situ* hybridisation (FISH) techniques first enabled the detection of DNA and RNA molecules in structurally preserved, fixed tissue sections (43, 324, 325). Oligonucleotides, complementary to a target nucleotide sequence, are labelled with single or multiple fluorophores. In turn, fluorescently labelled oligos bound to a target region can be observed using optical microscopy. Ultimately, the principles of FISH facilitated quantitative detection of mRNA at subcellular resolution (43, 324, 326). Here, the authors constructed a library, consisting of short single fluorophore-labelled oligos, against a single mRNA target to estimate the number of mRNA molecules in a single cell, screening up to 3 mRNA sequences in parallel.

To enable high-throughput spatial transcriptomic screening, Lubeck et al. first established the principles of sequential FISH (seqFISH), providing a strategy with theoretically whole transcriptome coverage (327, 328). In brief, multiple single fluorophore-labelled probes are used for mRNA labelling during a single hybridization round. By stripping probes and performing multiple rounds of hybridisation, the number of unique barcoding increases exponentially. Shah et al. demonstrated the efficacy of seqFISH for screening hundreds of genes at sub-cellular resolution, providing a novel insight into the spatial organisation and transcriptional heterogeneity of the mouse brain (329). The recent introduction of an additional fluorophore to sequential hybridisation allowed further scaling of seqFISH (seqFISH+) (**Figure 3** and **Table 2**) (266). This strategy avoids optical crowding by effectively diluting mRNA molecules into separate images. The result was a robust protocol for screening 10,000 genes in spatially resolved tissues, spanning thousands of cells (266). Here, the use of confocal microscopy for the seqFISH+ protocol provides a key advantage to facilitate wider adaption. A recent study by Lohoff et al. applied seqFISH to construct spatially resolved gene expression profiles for mouse organogenesis using a computational framework for the integration of spatially-resolved gene expression maps with scRNA-seq profiles of cell types in early mouse development (330, 331). In parallel, Chen et al. pioneered a multiplexed error-robust FISH (MERFISH) approach which combined error-corrected barcoded probes and sequential imaging to perform a multiplexed screen of hundreds of genes (**Figure 3** and **Table 2**) (267). Further MERFISH developments, such as the use of expansion microscopy, enabled quantification of thousands of genes in hundreds spatially resolved cells at a detection efficiency of ~80% (268). This high capture efficiency is a major advantage of MERFISH.

While the methods outlined above drove innovation in spatial transcriptomics, their relative infancy is accompanied by experimental and computational complexity, which currently provides a barrier to wide-spread adoption. Several commercially available platforms have been established to provide a standardised experimental framework. The Visium platform utilised NGS for deriving spatially resolved gene expression profiles (323, 332). Here, a tissue section of interest is deposited onto a slide, coated with uniquely barcoded arrays (barcode spacing permits 55um resolution). Following barcoding of captured mRNA molecules, cDNA libraries are subjected to high-throughput NGS and spatial deconvolution based on the unique barcoding. However, the current barcode spacing prevents interrogation of neighbouring cells. Here, *in situ* analysis can provide a complementary approach, allowing interrogation of a defined set of mRNA targets at spatial singe cell resolution (333–335).

Collectively, spatial transcriptomics technologies are currently in the developmental and early adaption phase. As a result, several key limitations persist. For instance, the tissue-dependent optimisation and sequential hybridisation rounds require significant experimental time, while the use of customised equipment also impacts implementation. However, increasing throughput and the desire to reach whole-transcriptome coverage will greatly increase imaging time and data complexity, making the most prominent limiting factor the development of robust analytical tools. To overcome the computational barrier, recent advances aim to address key unmet needs in data analysis and its scalability (336, 337).

Despite these challenges, several major advances have already been made using spatial transcriptomics, including studies in tumor heterogeneity and transcriptional changes in the microenvironment. In one study, Berglund et al. constructed a comprehensive spatial map of tumor and healthy prostate tissue biopsies from a prostate adenocarcinoma patient (322). The authors uncovered significant transcriptional differences between the tumor core and its periphery. Intriguingly, thorough interrogation of stromal and immune cell types, surrounding the primary tumor, facilitated the identification of heterogeneous gene expression networks in the tumor microenvironment (322). Spatial transcriptomics has also been applied for mapping the localisation of Cxcl12-abundant reticular cells in the bone marrow niche and for the characterisation of stromal cell heterogeneity in tumor microenvironments (338, 339). These and other studies demonstrate that the potential of spatial transcriptomics in deciphering tumor architecture, heterogeneity and microenvironments has been widely recognised. Beside its role in therapeutic discovery and disease pathology, spatially resolved gene expression profiles can become of paramount importance for monitoring therapeutic outcomes of cell therapies and identify evasion mechanisms in response to cell therapies. In addition, spatial characterisation post CAR T cell therapy could provide an insight into the impact of off-target effects on the function of proximal tissues. Similarly, spatial transcriptomics could aid in long-term monitoring of patients undergoing corrective gene therapies.

## Concluding Remarks

The past decade has produced an abundance of novel single-cell molecular tools, facilitating the unbiased screening of a wide array of molecular dimensions at unprecedented resolution. Unimodal sequencing technologies have proved particularly impactful in the first wave of wide-scale adoption, but more approaches have been focused on combining such techniques into multimodal screens to allow simultaneous capture of multiple molecular dimensions from the same cell. These technologies have allowed researchers to unpick the molecular mechanisms driving disease pathology at a scale not previously considered possible. Tissue and disease heterogeneity, previously masked in bulk sequencing approaches, are now routinely being explored at single cell resolution.

Techniques such as scRNA-seq have been widely adopted due to the production of robust experimental protocols and increasing consensus surrounding the computational approaches for quality control and data analysis. On the other hand, multimodal screens have not yet enjoyed similar uptake due to their reliance on high sequencing costs, advanced integrative computational tools and technical expertise. However, just as moving to single cells was a technical hurdle of 10 years ago, the research benefits derived from novel multimodal screening platforms will push the limits of discovery and accelerate technical development and method standardization. The next few years should see these technical and computational approaches streamlined to create reproducible protocols and standardised analytical pipelines to facilitate rapid adoption rates, as has occurred for scRNA-seq historically.

Concomitant with the technical challenges and need for standardization, the increased accessibility of single-cell technologies has exponentially increased the amount of data generated during these studies. This provides a unique opportunity to leverage the power of these studies by integrating datasets but also makes for substantial computing and processing challenges. Batch correction and data integration across experiments and different sequencing platforms are areas that will require particular attention and novel computational approaches for handling and analysing increasing amounts of data will be of paramount importance. Ultimately, the continuous technical improvements and aggregation of data could provide the foundation for a fully characterized reference atlas of the human body at single cell resolution. The drive towards such a resource is evident in the recently announced efforts to establish a common coordinate framework (CCF) for data collection and integration (340). In line with that, initiatives such as the Human Biomolecular Atlas Program and the CCF aim to provide a publicly available tool to help researchers map data from diseased states onto healthy single-cell datasets and provide a reference for the entire scientific community (340, 341).

A number of recent studies have clearly demonstrated the utility of these approaches in (1) understanding complex biological processes such as cell fate determination and immune response, (2) dissecting tissue and disease heterogeneity, and (3) stimulating innovative research aimed at developing novel therapeutics (342–344). Within the next decade, it is anticipated that an increasing number of patients across many disease types will be treated with gene and cell therapy. Using samples obtained from these growing patient cohorts, single-cell technologies will undoubtedly be used to answer essential questions related to the relationships between disease-causing cells, normal or corrected cell types, tumor-targeting lymphocytes such as CAR T cells, and endogenous immune populations. For autoimmune diseases such as type 1 diabetes where the risk of relapse is relatively high due to immunogenicity, this level of detail will be essential to finding new ways to increase treatment efficacy. Additionally, due to the relatively recent wider application of these therapeutics, only a limited number of gene or cell therapy clinical trial patients have been monitored for more than 10 years following treatment initiation (65, 84, 345–347). Depending on the stability of edited cells and the influence of other comorbidities, detailed studies using single-cell approaches may also become relevant during long-term follow up. As patients enter the later decades of life, the intersection of age-related and treatment-related abnormalities may present unique clinical challenges. Further refinements and innovations to single-cell profiling technologies have the potential to unlock and disentangle relationships between key drivers of disease phenotypes, leading to wider delivery of authentic personalised medicine.

## Author Contributions

DB and AC wrote and compiled the review. JR-L designed and created the figures. DK supervised the work and edited the manuscript. All authors contributed to the article and approved the submitted version.

## Funding

The DK laboratory is supported by an ERC Starting Grant (ERC-2016-STG–715371), an MRC-AMED joint award (MR/V005502/1), and the Bill and Melinda Gates Foundation (INV-002189). DB was supported by a Wellcome PhD Studentship and AC by the Bill and Melinda Gates Foundation (INV-002189).

## Conflict of Interest

The authors declare that the research was conducted in the absence of any commercial or financial relationships that could be construed as a potential conflict of interest.
